# Reflection on Five Months Rural District Hospital Posting of Final Year MBBS Students

**DOI:** 10.31729/jnma.8519

**Published:** 2024-03-31

**Authors:** Pragyan Basnet, Ritika Karki, Rupesh Acharya, Priyesh Lohani, Udaya Kumar Malla

**Affiliations:** 1 Patan Academy of Health Sciences School of Medicine, Lagankhel, Lalitpur, Nepal

**Keywords:** *medical education*, *medical school*, *rural health*

## Abstract

Patan Academy of Health Sciences has been sending its undergraduate medical students to rural postings aligning with the national health system of Nepal to produce competent and empathetic healthcare professionals as a part of its social accountability. One such rural posting is a 20-week-long district posting where students are posted at district hospital and district health office. We were final-year students posted at Gulmi district for this purpose in the year 2021/22. We learned the functioning of a district hospital along with different clinical skills. We also learned to use the district health information system and different qualitative tools in drafting district health reports and strategic planning under the guidance of the District Health Office. Such exposure of medical students is essential to develop competent and empathetic health professionals and similar provisions should be included in the undergraduate curriculum of other universities.

## INTRODUCTION

Medical schools have an important responsibility of ensuring good health in the community as a part of their social accountability.^1^ They are obliged to direct their service, education, and research towards the betterment of the community they have a mandate to serve.^2^ Realizing its importance, there have been several initiatives across the globe to modify the medical curriculum to incorporate social accountability.^3,4^

Patan Academy of Health Sciences (PAHS) is a medical school from Lalitpur, Nepal established in 2008 with a mission to serve the health needs of rural communities of Nepal.^5^ To meet this mission and fulfill its social accountability, it has been preparing medical students, and future doctors, to work in rural communities of Nepal aligning with the national health system.^5^ PAHS has adopted community-based Based Learning and Education (CBLE) as an innovative teaching-learning system where students are sent to different rural community postings during their undergraduate course, MBBS, to learn about the health system of Nepal and adapt to work in rural Nepal.^5^ In these postings students are exposed to different levels of the national health system of Nepal ranging from female community health volunteer (FCHV) level to district level.

There is a 6-month long district level posting in the final year of Bachelor of Medicine and Bachelor of Surgery (MBBS) curriculum which is the longest among all these rural postings at PAHS.^5^ This posting is further broken down into 5 months district hospital posting and 1-month of district health office posting. Due to academic delays of COVID-19 pandemic, this posting was cut short to 20 weeks (17 weeks at district hospital and 3 weeks at the district health office). PAHS students are posted at Gorkha, Kapilvastu and Gulmi Districts. In this article, we would like to share our reflections as PAHS MBBS students in a 20-week-long posting at District Hospital and District Health Office of Gulmi district during our final years in the year 2021/2022. The experience of PAHS students may be helpful feedback to other medical colleges and universities of the country to implement similar programs in their MBBS and other health sciences curricula.

## DISTRICT HOSPITAL POSTING

The posting at the district hospital was a clinical posting under the supervision of a medical generalist. The doctor's team at the district hospital consisted of two consultant medical generalists, an MD in General Practice and Emergency Medicine (MDGP) final-year resident from PAHS, and medical officers. We also learned from experienced healthcare professionals consisting of nurses, auxiliary nurse midwives (ANM), and health assistants (HA). We divided ourselves into groups of 2 each and rotated in the emergency department (ED), outpatient department (OPD), and inpatient (indoor) department.

The hospital had good patient flow owing to the provision of national health insurance along with free or subsidized treatment costs from the government. Unlike Patan Hospital, a tertiary care center, this hospital was functioning in resource-limited settings. In the ED, the first responders were HA and ANM who were responsible for the primary survey. They would inform the duty doctor (medical officers), the first on-call, depending on the severity of the case. The duty doctor would then guide the first responders and inform the second on-call medical generalist if necessary. There was only one duty doctor posted at the ED and indoors for 24 hours which sometimes made it difficult for the doctor to handle all the cases.

We observed and assisted hospital staff in managing the cases. In the OPD, under the supervision of doctors, we observed and assisted in history taking, examination, investigation, diagnosis, and management of the patient. In the indoor, we learned to monitor the patient, write progress notes, and manage the patients. In this hospital, we got a chance to observe and assist cases of normal delivery and observe cesarean sections as well. As per the PAHS curriculum, we were assigned to learn certain sets of clinical skills like cannulation, basic suturing skills, assisting in normal delivery, etc., and to complete evaluation after completion of each task from the local faculty medical generalist. We found that it was easier to learn clinical skills in the setting of a district hospital than in Patan Hospital as patient flow was high and only 6 of us were posted at this center. We used to discuss medical cases in morning handover under the supervision of our local faculty which created a good academic environment in the hospital.

Besides clinical skills, we also learned the challenges of working at rural hospitals. Due to the unavailability of Computed Tomography (CT) scans, patients had to be referred to Palpa which would take 3-4 hours by road. There was a lack of sufficient health workers and the hospital needed more doctors and trained health professionals.

## DISTRICT HEALTH OFFICE POSTING

In district health office posting, each student was assigned an in-depth report on various public health issues of the district. The topics were finalized by joint efforts of faculty and students after communicating with local health officials. The topics during our posting were intra-uterine contraceptive Devices (Family Planning), Tuberculosis, Anti-Retroviral Treatment Program, etc. where we analyzed a year's trend of services provided by the health office. Then each group was assigned a topic for the overall district health report and strategic planning. We collected qualitative data for our report using tools like key informant interviews (KII) and In-depth Interviews. We learned and then collected quantitative data using the District Health Information System (DHIS-2). The topic of strategic planning for one of the groups was to ensure a tuberculosis-free Gulmi. We collected the necessary data and made a strategic plan after analyzing the status of tuberculosis in Gulmi. The topic of strategic planning for another group was to increase coverage of COVID-19 vaccination where we analyzed the status of the COVID-19 vaccination program and suggested strategies for 100% coverage of the vaccine in Gulmi. We presented the findings in the presence of staff and officials of the District Hospital and District Health Office. The health office staff gave us positive feedback for our reports saying that it helped them as an important resource for carrying out future health activities in the district.

## LEARNINGS FROM THE LOCAL COMMUNITY

One of the important objectives of this posting was to familiarize medical students with the local communities. Our hostel was in a rented flat in one of the local's houses a few meters away from the hospital. This provided us with sufficient opportunities to interact with local people and learn about their health needs. We also got a chance to interact with rural healthcare workers of our country and were motivated by their dedication to serving despite many challenges. We celebrated local festivals with community people and this was a great opportunity to learn from cultural diversity. Besides we were also introduced to the difficult geography of the hilly region of Nepal and how it challenges people's access to healthcare. In this way, we were interacting with the community whom we intend to serve in the future as doctors.

## WAY FORWARD

District posting of final-year MBBS students serves both students and the community. The District Hospital gets benefits from students as enthusiastic health volunteers and the District Health Office gets benefits from student research and their reports. Early exposure to medical students in rural settings helps them to work in rural communities as doctors and also encourages them to stay within the country. It is essential to develop technically competent as well as empathetic physicians. This exposure also helps during government bonding of physicians in rural areas. A similar curriculum should be implemented in other medical colleges of the country to develop competent and empathetic physicians.
